# A Regional Analysis of the Progress of Current Dog-Mediated Rabies Control and Prevention

**DOI:** 10.3390/pathogens11101130

**Published:** 2022-09-30

**Authors:** Koji Kanda, Ananda Jayasinghe, Chandrika Jayasinghe, Takahiko Yoshida

**Affiliations:** 1 Department of Social Medicine, School of Medicine, Asahikawa Medical University, Asahikawa 078-8510, Japan; 2 Department of Community Medicine, Faculty of Medicine, University of Peradeniya, Peradeniya 20040, Sri Lanka; 3 Department of Medicine, Faculty of Medicine, University of Peradeniya, Peradeniya 20040, Sri Lanka

**Keywords:** dog-mediated rabies, elimination, dog vaccination, surveillance, Asia, Africa

## Abstract

This study aimed to assess the current progress of dog-mediated rabies control and the level of political commitment among 88 rabies-endemic countries and to provide further recommendations for the elimination of dog-mediated rabies by 2030. A correlational study was conducted using data and relevant regulations from the websites of international organizations and NGOs. In general, rabies was yet to be considered a priority disease and only one out of five countries and territories has prepared a national strategic plan for rabies control and prevention. Likewise, scores of dog-mediated rabies control indicators such as dog vaccination rate and the number of post-exposure prophylaxis per 1000 people remained minimal. There were also regional differences in preparation for dog-mediated rabies control and progress towards elimination. In particular, more efforts are needed for Pan-African Rabies Control Network (PARACON) member countries. In order to meet the goal of global dog-mediated zero rabies by 2030, both dog-mediated rabies control activities such as dog vaccination and strong political commitment should be strengthened and promoted in all rabies-endemic regions of the world.

## 1. Introduction

Lyssavirus rabies, the family *Rhabdoviridae*, genus *Lyssavirus*, results in almost 100% fatality, with a year 2015 estimation of approximately 59,000 deaths worldwide [1]. There are more than 15 million human exposures to rabies, mostly in Asia and Sub-Saharan Africa, resulting in 3.7 million Disability-Adjusted Life Years (DALYs) and a huge economic loss of USD 8.6 billion per year [2]. The international community has now called for the world to be canine rabies free by 2030 (Zero by 30); specifically, no indigenously acquired dog-mediated rabies cases among humans, to be achieved by the year 2030. At present, only selected countries, including Western Europe, North America, Japan, and some Latin American nations enjoy a rabies-free status [1].

Zero by 2030 was initiated through the collaboration of four different organizations: the World Health Organization (WHO), the World Organization for Animal Health (OIE), the Food and Agriculture Organization of the United Nations (FAO), and the Global Alliance for Rabies Control (GARC) [3]. Their strategies to reach Zero by 30 condense the societal changes into three objectives: (1) To effectively use vaccines, medicines, tools, and technologies; (2) To generate, innovate, and measure impact; and (3) To sustain commitment and resources [3]. The first objective leads to the reduction of human rabies risk. Rabies is preventable by scientifically-proven interventions, such as awareness-raising, post-exposure prophylaxis (PEP), and mass dog vaccination [3]. Various efforts of raising awareness contribute to people’s understanding of rabies as well as proper preventive measures, including avoiding unnecessary animal bites [4,5,6,7,8]. PEP should be administered without delay according to the type of exposure. Particularly, scheduled vaccinations are required for WHO category II exposures of nibbling of uncovered skin or minor scratches or abrasions without bleeding, and vaccinations plus rabies immunoglobulin (RIG) are recommended for category III of single or multiple transdermal bites or scratches [1]. Though often costly, it is almost 100% effective, even when the exposure is severe, and therefore its secured access prevents the onset of rabies-related symptoms among people of every class, including the world’s most underserved groups [1,3,9]. Mass dog vaccination is an effective way to stop the dog-to-dog or dog-to-human transmissions of rabies viruses by at least 70% of vaccination coverage [3,10,11,12]. Several studies have shown a clear negative relationship between rabies incidence and vaccination coverage over the decades [2,13,14,15,16]. These indicators for rabies control should be monitored periodically to evaluate their effectiveness and achievements towards the final goal of zero rabies. The second objective of generating, innovating, and measuring impact is subcategorized into “policy, guidance, and governance” and “reliable data”. Particularly, providing the necessary support and effective decision-making are expected through a clear, solid political framework and reliable data collection on human and animal rabies cases, animal bites, and laboratory testing, as well as on the completion of the above interventions at global, regional, and national levels. However, a paucity of integrated data has been reported in many parts of the world due to the poor understanding of the importance of surveillance, the variety of its system in each country and region, and/or limited staff and institutional capacity for testing, including a partial collaboration between human and animal sectors [17,18,19]. Inadequate data management has become a barrier to the development and implementation of effective rabies control policies and strategies, despite extensive control efforts such as canine vaccination and animal birth control [18,20]. The third objective is mainly focused on key stakeholders’ consistent engagement in rabies elimination and effective use of financial and other resources. Effective advocacy for stakeholders promotes their continuous involvement and provides adequate resources for rabies elimination. The multidisciplinary One Health approach can support the reduction of human rabies cases as well as prompt response to suspected rabies cases [21,22]. Due to the neglected nature of the disease, however, there is still limited concern for their engagement and, therefore, in national and regional sectors, maintaining an adequate level and volume of rabies control and prevention activities is problematic.

To effectively monitor the progress towards the final goal of Zero by 30, it is essential to assess the available data periodically at the global level. Regional collaborative anti-rabies networks have been established in recent years, namely the Asian Rabies Control Network (ARACON); the Middle East, Eastern Europe, Central Asia, and North Africa Rabies Control Network (MERACON); and the Pan-African Rabies Control Network (PARACON) [23,24]. As of June 2021, 88 rabies-endemic countries belong to the networks and periodically provide the latest data related to rabies control at the GARC website (https://rabiesalliance.org/ accessed on 23 June 2021). Though the data are far beyond comparable due to their incompleteness, regular monitoring is still essential at the global level. Many countries regularly report human rabies cases and other additional rabies-related information, but there is no such integrated analysis of the current progress of dog-mediated rabies elimination involving epidemiological data, anti-rabies practices, the nation’s financial contribution, and political commitment. Providing the evidence according to the Zero by 30 strategy will directly promote the commitment towards elimination and provide adequate resources for anti-rabies activities.

Therefore, the purpose of this study is to explore the latest, available data to assess the current progress of rabies control by region and provide further recommendations for elimination.

## 2. Results

### 2.1. Descriptive Analysis toward Zero by 30 Targets

A total of 88 rabies-endemic countries were involved in the analysis. ARACON consisted of 15 rabies-endemic countries in East, Southeast, and South Asia, including Bangladesh, Bhutan, Cambodia, China, India, Indonesia, Laos, Malaysia, Myanmar, Nepal, Pakistan, Philippines, Sri Lanka, Thailand, and Viet Nam. Another 25 countries belonged to MERACON, which were Afghanistan, Algeria, Azerbaijan, Croatia, Egypt, Georgia, Iran, Iraq, Jordan, Kazakhstan, Lebanon, Libya, Morocco, Palestine, Poland, Qatar, Romania, Russia, Serbia, Tajikistan, Tunisia, Turkey, Ukraine, Uzbekistan, and Yemen. The rest of the 48 countries and territories belonged to PARACON, which included all sub-Sahara African countries and three territories (Somaliland, Western Sahara, Zanzibar), except for three rabies-free islands (Carbo Verde, Mauritius, Seychelle).

**Figure 1:**
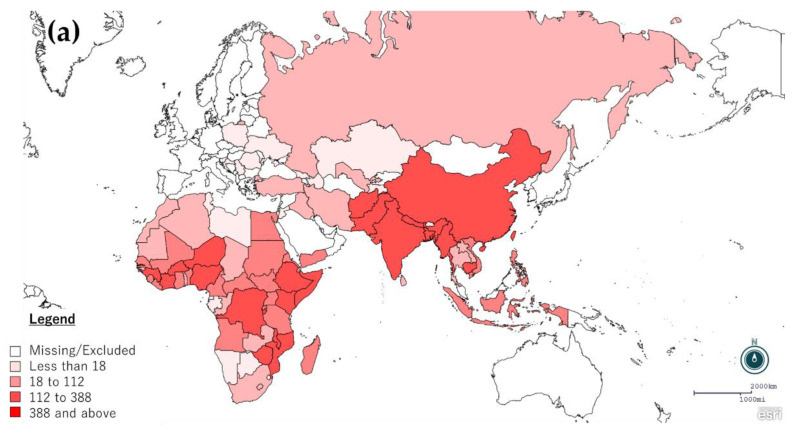

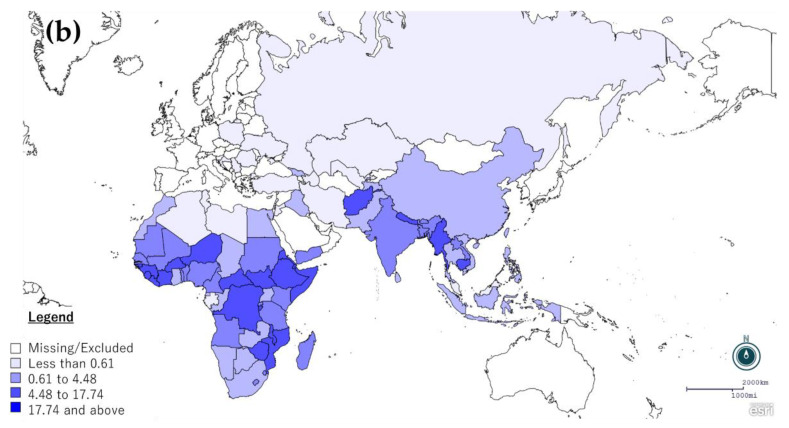
Distribution of (**a**) annual human rabies deaths and (**b**) rabies mortality rate per 1,000,000 population by each country, as of June–July 2021. Data were obtained from the Global Alliance for Rabies Control website (https://rabiesalliance.org/ accessed on 23 June 2021), under the sections of “Country Status” and “Rabies Burden Estimates” of each country in either of three regional networks.

The current human rabies epidemiology among the above 88 countries and regions are described in Figure 1, which includes the data available from the GARC website (https://rabiesalliance.org/ accessed on 23 June 2021) as of June–July 2021. Data were obtained from the sections of “Country Status” and “Rabies Burden Estimates” of each country’s website under either of the three regional networks. The highest number of rabies deaths was recorded in India (20,847), followed by China (6003), and DR Congo (5579). The countries with annual deaths exceeding 1000 were Myanmar (4552), Ethiopia (2771), Afghanistan (1768), Nigeria (1637), Mozambique (1326), Niger (1169), Somalia (1154), and Nepal (1044) (Figure 1a). Due to the volume of the total population, there were a greater number of annual rabies deaths in ARACON countries than in others. The total number of worldwide rabies deaths in the available data reached 58,946. Particularly, only four ARACON nations (China, India, Myanmar, and Nepal) reached 92.3% (32,446/35,146) of entire human rabies deaths among all ARACON nations. On the other hand, rabies mortality rates were more severe in African countries (Figure 1b). Twenty-five (25) out of 48 countries and territories in Africa reached at least 10 rabies cases per 1,000,000 population, in comparison with five out of 15 Asian counterparts. Particularly, African countries with less than 1,000,000 population affected by rabies deaths included Eritrea (104.66), Central African Republic (47.84), Liberia (45.78), Sierra Leone (38.53), and Guinea-Bissau (37.48). These numbers are much larger than in populous countries, such as India (15.26) and China (4.19). In MERACON countries, most countries seem to be well controlled of rabies transmission, except for Afghanistan (1768 deaths and a mortality rate of 46.67 per 1,000,000 population). Approximately half of the counties recorded 10 or fewer human rabies deaths, including no cases in four countries (Croatia, Poland, Qatar, Serbia) and only one case in another three countries (Jordan, Lebanon, Romania).

**Figure 2:**
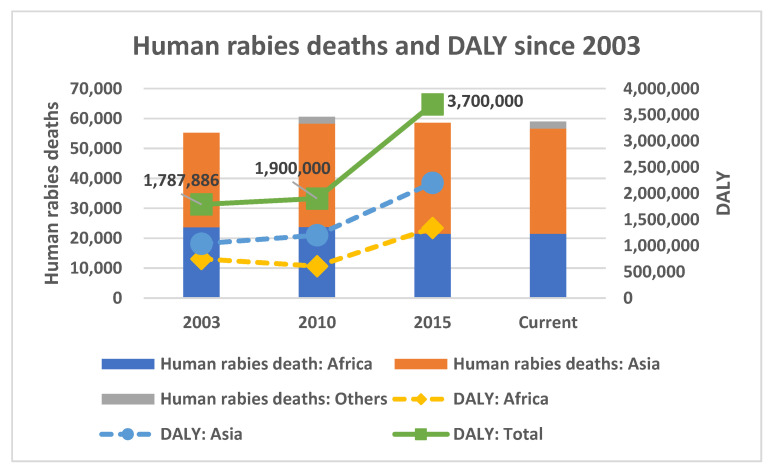
Human rabies deaths and DALY since 2003. A total DALY in 2003 included Africa and Asia only. (Sources: [1,2,25,26]).

Figure 2 describes the latest history of worldwide human rabies deaths and DALY. Despite the initiation of Zero by 30, unfortunately, there has been no significant change in rabies deaths over the decades [1,2,25,26]. It has remained the same that most human rabies deaths occurred in Africa and Asia. Instead, the DALY estimate was largely increased in 2015. It nearly doubled, both in Asia and Africa [1,2,25]. On the other hand, there were limited data on the cost of rabies control and the dog population. The 2015 estimate of the rabies control cost was USD 8.6 billion dollars, which had increased from USD 6 billion dollars in 2010 [1,25]. Among them, approximately USD 1.5 billion dollars was spent in Asia during the period 2010–2015 [1,25]. It was more difficult to observe the data on the canine population at the global level. Knobel et al. (2005) estimated the human-to-dog ratio of 12.3 in Africa and 9.5 in Asia in the early 2000s [26].

Table 1 shows the interim results of the societal change toward rabies elimination by three regional networks. As mentioned in Figure 1, the average number of human rabies deaths was significantly higher in PARACON countries (21.5) than in ARACON (13.7) and MERACON (2.5) counterparts (*p* < 0.001). Conversely, the dog vaccination coverage was the lowest in PARACON (4.1), among the three regional groups (*p* < 0.001). In most of them (40 out of 48), it has not reached 1%, or the data were not reported. Only South Africa reported 63% coverage, but the nation recorded 42 human rabies deaths at the same time. None of the 88 countries and regions has met the target coverage of 70%. Besides South Africa in PARACON, the highest coverage in each network was Poland (62.4%) in MERACON and Thailand (49.5%) in ARACON. No human rabies deaths were observed in Poland, but Thailand suffered 62 human rabies deaths. In ARACON, the dog vaccination coverage was also negligible in Myanmar (0.6%) and Nepal (0.17%) and remained up to 15% in India. The average cost of rabies prevention and control was USD 1.2. There was no significant difference between ARACON and PARACON, but 20 PARACON countries and territories (41.7%) spent USD 1 or less for rabies control and prevention per population. The highest cost was spent in South Sudan (USD 8.45), followed by Myanmar (4.98), Eritrea (3.79), and Djibouti (3.01). The mean number of people who receive PEP per 1000 population was the highest in ARACON (6.6). Limited access to PEP in PARACON was evident (0.9, *p* = 0.001). Despite the highest number of rabies deaths among the three networks, the total number of PEP per 1000 population was one or below in three-fourths of countries and territories.

Regarding national policy and regulations, only one-third of 88 countries and territories treated rabies as a top five priority diseases, and approximately half of them designated rabies as a notifiable disease. A national rabies control strategy is currently available or being developed only in 16 and 17 countries, respectively. The mean Stepwise Approach towards Rabies Elimination (SARE) Score was 1.5, indicating that small-scale rabies control programs are introduced and a national control strategy is being prepared. Among three regional networks, ARACON seemed to have a stronger political commitment and to provide a rabies control environment compared to other networks. Although there was a large amount of missing information among MERACON member countries, regulations and readiness towards rabies elimination among ARACON countries were more advanced than those among PARACON countries and territories (*p* < 0.001). Less than half of the nations organized a One Health working group or rabies task force.

### 2.2. Correlational Analysis among Indicators of Rabies Prevention and Control

**Figure 3:**
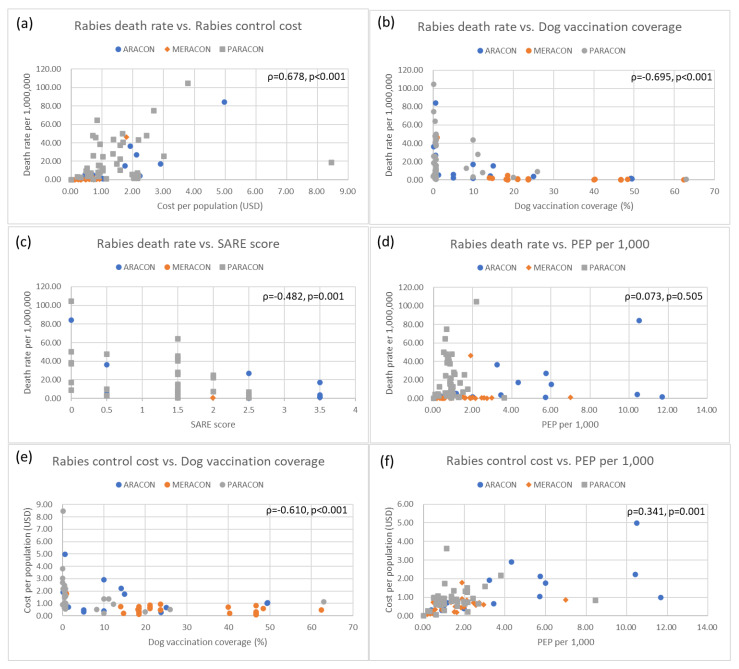
Correlations among major rabies control indicators by three regions: (**a**) Rabies death rate vs. rabies control cost; (**b**) Rabies death rate vs. dog vaccination coverage; (**c**) Rabies death rate vs. SARE score; (**d**) Rabies death rate vs. PEP per 1000; (**e**) Rabies control cost vs. dog vaccination coverage; and (**f**) Rabies control cost vs. PEP.

Figure 3 depicts the correlations among epidemiological indicators of human rabies death rate, dog vaccination rate, rabies control cost, SARE score, and the number of PEP. There were strong correlations between rabies death rate and rabies control cost (Spearman’s rho (ρ) = 0.678, *p* < 0.001), rabies death rates and dog vaccination rate (ρ = −0.695, *p* < 0.001), rabies death rate and SARE score (ρ = −0.482, *p* < 0.001), rabies control cost and dog vaccination coverage (ρ = 0.610, *p* < 0.001), and rabies control cost and the number of PEP per year (ρ = 0.341, *p* < 0.001). In summary, the rabies death rate decreased when the dog vaccination coverage and/or SARE score increased, and the rabies control cost increased when the rabies death rate and/or PEP per 1000 increased. However, the rabies control cost decreased when the dog vaccination coverage increased.

A regional difference was also observed among the indicators of interest. The rabies death rate decreased as the dog vaccination rate increased among ARACON countries (ρ = −0.748, *p* < 0.001), but their relationship was not statistically significant among other networks. Likewise, there was a statistically negative relationship between the rabies death rate and SARE score among PARACON members (ρ = −0.379, *p* = 0.032) but no such cases in other counterparts. In terms of rabies control cost, it was likely that the increasing cost resulted in the higher rate of rabies death and/or PEP in all three networks, though there were no statistically significant relationships between the cost and death in MERACON (ρ = −0.392, *p* = 0.052) and the cost and PEP in PARACON (ρ = −0.259, *p* = 0.086). The dog vaccination rate was also negatively associated with the cost only in PARACON countries (ρ = −0.502, *p* = 0.001).

Table 2 shows the relationships between epidemiological indicators mentioned above and the qualitative counterparts of legal and political interventions. In general, the countries that identified rabies as a priority disease seemed to record a lower rabies death rate and control cost per population (χ^2^ = 5.951, *p* = 0.015), and a higher amount of dog vaccination coverage (χ^2^ = 10.321, *p* = 0.001). In addition, the nation having or preparing a national strategy for rabies control was more likely to provide PEP (χ^2^ = 13.273, *p* < 0.001). The higher SARE score resulted in a lower rabies death rate (χ^2^ = 6.116, *p* = 0.013).

## 3. Discussion

In 2015, the world called for action towards the goal of zero human dog-mediated rabies deaths by 2030. A global strategic plan suggested three main objectives for combating the disease: “To effectively use vaccines, medicines, tools, and technologies”, “To generate, innovate, and measure impact”, and “To sustain commitment and resources” [3]. To meet its ultimate goal, a three-phase approach of “start-up”, “scale-up”, and “mop-up” is currently being pursued, and the first phase ended in 2020 [3]. Therefore, it would be essential to assess the country’s progress towards the elimination at this stage so that the latest information regarding rabies-related policy, regulations, and epidemiological data on rabies death and preventive measures among rabies-epidemic countries is reviewed.

Our collected data showed that the worldwide annual human rabies deaths remained analogous to the initial estimate of 59,000 in 2015 [2]. This is likely due to delayed data updates and poor surveillance as well as inadequate rabies control and prevention activities in many countries. Limited data updates and poor surveillance are considered administrative barriers to the health and animal sector, along with inadequate capacity of rabies diagnostic laboratories and lack of political interest in rabies control [20]. The collection and publication of accurate epidemiological data will not only allow us to understand the current status of rabies elimination in each country but also to evaluate the effectiveness of the various measures that have been implemented to date. Appropriate data collection and management is the provision of effective guidance for decision making for the final goal, and this then meets the second pillar of zero rabies strategies [1]. In fact, rabies-related data have been visualized and used for policy makings through collaboration among various departments in successful rabies-controlled countries and regions [27]. In South America, where canine rabies is being eliminated, thorough data collection and the accompanying appropriate disclosure of information have provided a steppingstone to rabies elimination in a short period of time [28]. Throughout history, raw data can be observed until today as a result of thorough surveillance in a country where domestic rabies has been eliminated for more than a half-century [29,30]. A robust surveillance system ensures the follow-up of continuous anti-rabies activities after elimination, such as pet dog registration and vaccination coverage [29,31]. Fortunately, there is a region-wide movement to promote data visualization, such that PARACON has begun operating a system to collect and manage data within the region [23]. Although the data have not yet been fully disclosed, the system should be strengthened in the entire region to scale up the elimination activities and meet the final goal of no dog-mediated rabies. Similarly, the implementation of anti-rabies measures should also be monitored. The data obtained in this study were canine vaccination coverage, rabies control cost, and the number of people who received PEP. Particularly, the incidence of PEP is considered to be one of the minimum epidemiological indicators to be provided by rabies surveillance and a proxy for suspected and confirmed exposure to rabies [1]. However, as with the surveillance data above, it cannot be said that accurate information has been widely disseminated in public, so that the actual situation is not yet clear. This is a huge barrier to the distribution of resources related to rabies control and therefore should be improved accordingly. The same is true for canine vaccination coverage, where effective collaboration between the human and animal sectors is key to collecting and disseminating proper information for future elimination strategies. Lack of information regarding canine vaccination coverage may include the missing practice of pet dog registration, resulting in the fact that the total number of dogs is not being fully recognized. For this reason, it is essential to establish an appropriate surveillance system, and the health sector and the animal sector in each country should collaborate to maintain and manage the database.

Our deep statistical analysis indicated that the rabies death rate was strongly associated with variables related to rabies control. We demonstrated that the rabies death rate was negatively associated with the canine vaccination rate. This was additional proof of previous outcomes in countries where intensive dog vaccination contributed to the reduction of dog-mediated rabies [3,13]. The countries where rabies was considered a priority disease showed lower human rabies death rates, higher dog vaccination coverage, and decreased cost of rabies control per population. In addition, implementing the national strategy toward rabies elimination would induce more PEP utilization. These outcomes imply that prioritization of the disease would positively affect rabies control and prevention. However, our current information revealed that the dog vaccination rate was generally low worldwide. This might be due to the fact that, as with epidemiological data, vaccination data are not regularly updated, but it is also inferred that actual dog vaccination programs are not widely disseminated in the community.

For policy and regulations, a limited number of countries provided national anti-rabies guidelines, legislation, and/or strategies at this stage. Although more than half of the 88 countries and territories regulated both human and animal rabies as notifiable diseases, it was only in 12 countries (13.6%) that all of the five policy-related statements listed in Table 1 included rabies in the top five priority diseases, notifiable both in human and animal cases, as well as there being the operation of a national strategic plan towards elimination and a One Health working group/Rabies task force (Bangladesh, Benin, Bhutan, Cambodia, Cote d’Ivoire, Indonesia, Kenya, Namibia, Philippines, Sierra Leone, Thailand, Viet Nam). The “Zero by 30” initiative states that each country is requested to prepare robust, budgeted, effective, and sustainable national rabies elimination plans following a One Health approach during the first “start-up” phase, and 29 out of 100 rabies-endemic countries are supposed to meet the goal of elimination by 2020 [3]. However, only Mexico in Latin America has received WHO validation on rabies elimination so far, and no countries and territories in our database have reached the level of zero dog-mediated rabies. Though it might be inappropriate to evaluate the progress based on the data used in the analysis due to the anonymity of the released date of the data as well as there being a large amount of missing information in MERACON nations, anti-rabies policy and regulations should be immediately prepared in all endemic countries, particularly in the PARACON group, to take further actions towards rabies control and elimination. To do this, strong political commitment is essential in all member countries. Recently, this has been demonstrated in Latin America, where canine rabies was politically recognized as a public health issue and subsequently sustainable rabies control activities were successfully implemented [32].

The limitations in this study include the quality of the dataset. We obtained the data from various sources, including the latest literature and a worldwide rabies control website. While these sources are reliable, they are not always the most up-to-date. In particular, worldwide dog-mediated rabies control strategies should heavily focus on the reduction of human rabies deaths in Asia and Africa, but much of the latest rabies data in MERACON were missing and did not provide any indication of the current situation. Additional important information for developing rabies control strategies, such as the current canine population, has not yet been compiled at the global level. Therefore, regular collection and analysis of epidemiological data in regions, countries, and the world will lead to the early achievement of dog-mediated rabies elimination.

## 4. Materials and Methods

This was a descriptive and correlational study assessing current dog-mediated rabies control and prevention, epidemiology, the status of program implementation, and political commitment among 88 rabies-endemic countries and territories in Asia, the Middle East, Eastern Europe, and Africa. Data were obtained from the latest literature and the websites of international organizations and NGOs, mainly including the GARC website (https://rabiesalliance.org/ accessed on 23 June 2021), under the sections of “Country Status” and “Rabies Burden Estimates” of each country in either of three regional networks. They included each country’s population, number of human rabies deaths, dog vaccination coverage rate, cost of rabies control, number of annual PEP treatments, and stepwise approaches towards rabies elimination (SARE) score as numerical values. SARE is a practical planning, monitoring, and evaluation tool to guide, develop, and refine rabies control programs, and its score shows the progress of the program, from 0 as no information on rabies or endemic to 5 as free from dog-transmitted rabies [33]. Based on these criteria, Stage 2 involves strategic planning and Stage 3 reaches the stage of rabies control. Regarding the assessment of legal and political commitment, we also collected the following qualitative information: the administrative status of rabies as one of the top five priority diseases and notifiable diseases in humans and animals, the development of a national strategy, and the involvement of a One Health working group or rabies task force. These qualitative indicators included a yes–no value, except for the one regarding national strategy development, which was recorded as either “available”, “being developed”, or “no”. Data were collected from 23 June to 29 July 2021.

Both bivariate and multivariate analyses were performed to depict the current conditions of rabies control and prevention by three regional anti-rabies networks: ARACON, MERACON, and PARACON. Because the obtained data were not normally distributed, we performed a non-parametric test to analyze the differences between the indicators of interest. During the analysis, correlations of each numerical indicator were also obtained by Spearman’s Rho and compared with the tendency and progress of the government’s policy towards rabies control by either Mann–Whitney’s U test or Kruskal–Wallis test. A p-value less than 0.05 was considered statistically significant. All data were analyzed using EpiInfo^TM^ Version 7.2.2.6 (US Centers for Diseases Control and Prevention, Atlanta, GA, USA).

## Figures and Tables

**Table 1:** Descriptive statistics towards Zero by 30 targets among three regional networks, as of June–July 2021.

	ARACON (n = 15)		MERACON (n = 25)		PARACON (n=48)		TOTAL (n = 88)		*p*-Value
**Intervention:**									
Death rate per 1,000,000	13.7	(0–4.2)	2.5	(0–46.5)	21.5	(0.5–104.7)	14.5	(0–04.7)	<0.001
Dog vaccination coverage (%)	15.0	(0.2–49.5)	29.1	(1–62.4)	4.1	(0.1–63.0)	13.3	(0.1–63.0)	<0.001
Cost per population (USD)	1.4	(0–5.0)	0.5	(0–1.8)	1.5	(0–8.5)	1.2	(0–8.5)	<0.001
PEP per 1000	4.4	(0–11.7)	1.6	(0–7.0)	0.9	(0–3.6)	1.7	(0–1.7)	0.001
**Policies:**									
Top five priority diseases (%)	12	(80.0%)	1	(4.0%)	16	(33.3%)	29	(33.0%)	<0.001
Notifiable in human (%)	10	(66.7%)	3	(12.0%)	32	(66.7%)	45	(51.1%)	<0.001
Notifiable in animals (%)	14	(93.3%)	3	(12.0%)	33	(68.8%)	50	(56.8%)	<0.001
National strategy									
Available (%)	9	(60.0%)	0	(0.0%)	7	(14.6%)	16	(18.2%)	<0.001
Being developed (%)	4	(26.7%)	2	(8.0%)	11	(22.9%)	17	(19.3%)	
One Health working group or Rabies task force (%)	9	(60.0%)	3	(12.0%)	22	(45.8%)	34	(38.6%)	0.003
Stepwise Approach towards Rabies Elimination (SARE) Score	2.1	(0–3.5)	1.8	(1.5–2.0)	1.2	(0–2.5)	1.5	(0–3.5)	0.043

**Table 2:** Relationships among rabies control indicators and policies.

	Death Rate Per 1,000,000		Dog Vaccination Coverage		Cost Per Population		PEP Per 1000	
	Number	*p*-Value	Number	*p*-Value	Number	*p*-Value	Number	*p*-Value
Disease priority								
Yes	12.165	0.015	11.782	0.001	1.015	0.015	2.345	0.299
No	45.686		0.395		2.478		3.180	
Notifiable in human								
Yes	16.940	0.856	9.624	0.241	1.106	0.121	1.734	0.645
No	21.310		3.237		1.960		3.172	
Notifiable in animals								
Yes	18.079	0.586	9.055	0.427	1.222	0.882	1.999	0.092
No	4.913		0.650		1.303		0.409	
National strategy								
Available/Being developed	17.987	0.953	10.981	0.526	1.374	0.284	2.941	<0.001
No	16.116		4.982		1.028		0.689	
One Health								
Yes	14.495	0.273	9.770	0.198	1.171	0.846	1.635	0.705
No	23.486		6.720		1.332		2.519	
SARE score								
Less than 2.0	22.943	0.013	6.135	0.194	1.325	0.488	2.143	0.165
2.0 and above	8.045		15.249		1.128		2.253	

## Data Availability

All data generated or analyzed during this study are included in this published article.

## References

[1] World Health Organization (2018). WHO Expert Consultation on Rabies.

[2] Hampson K., Coudeville L., Lembo T., Sambo M., Kieffer A., Attlan M., Barrat J., Blanton J.D., Briggs D.J., Cleaveland S. (2015). Estimating the global burden of endemic canine rabies. PLoS Negl. Trop. Dis..

[3] World Health Organization, Food and Agriculture Organization of the United Nations, World Organisation for Animal Health, Global Alliance for Rabies Control Partners for Rabies Prevention (2018). Zero by 30: The Global Strategic Plan to End Human Deaths from Dog-Mediated Rabies by 2030.

[4] Kanda K., Obayashi Y., Jayasinghe A., de S. Gunawardena G.S.P., Delpitiya N.Y., Priyadarshani N.G.W., Gamage C.D., Arai A., Tamashiro H. (2015). Outcomes of a school-based intervention on rabies prevention among school children in rural Sri Lanka. Int. Health.

[5] Matibag G.C., Ohbayashi Y., Kanda K., Yamashina H., Bandula Kumara W.R., Gamini Perera I.N., Niranjala De Silva D.D., Gunawardena G.S.P.D.S., Jayasinghe A., Ditangco R.A. (2009). A pilot study on the usefulness of information and education campaign materials in enhancing the knowledge, attitude and practice on rabies in rural Sri Lanka. J. Infect. Dev. Ctries..

[6] Shen J., Rouse J., Godbole M., Wells H.L., Boppana S., Schwebel D.C. (2017). Systematic Review: Interventions to Educate Children About Dog Safety and Prevent Pediatric Dog-Bite Injuries: A Meta-Analytic Review. J. Pediatr. Psychol..

[7] Duperrex O., Blackhall K., Burri M., Jeannot E. (2009). Education of children and adolescents for the prevention of dog bite injuries. Cochrane Database Syst. Rev..

[8] Amparo A.C.B., Mendoza E.C.B., Licuan D.A., Valenzuela L.M., Madalipay J.D., Jayme S.I., Taylor L.H. (2019). Impact of Integrating Rabies Education Into the Curriculum of Public Elementary Schools in Ilocos Norte, Philippines on Rabies Knowledge, and Animal Bite Incidence. Front. Public Health.

[9] World Organisation for Animal Health, World Health Organization, Food and Agriculture Organization of the United Nations (2015). Rabies: Rationale for Investing in the Global Elimination of Dog-Mediated Human Rabies.

[10] Cleaveland S., Kaare M., Tiringa P., Mlengeya T., Barrat J. (2003). A dog rabies vaccination campaign in rural Africa: Impact on the incidence of dog rabies and human dog-bite injuries. Vaccine.

[11] Coleman P.G., Dye C. (1996). Immunization coverage required to prevent outbreaks of dog rabies. Vaccine.

[12] Davlin S.L., Vonville H.M. (2012). Canine rabies vaccination and domestic dog population characteristics in the developing world: A systematic review. Vaccine.

[13] Harischandra P.A.L., Gunesekera A., Janakan N., Gongal G., Abela-Ridder B. (2016). Sri Lanka takes action towards a target of zero rabies death by 2020. WHO South East Asia J. Public Health.

[14] Darkaoui S., Cliquet F., Wasniewski M., Robardet E., Aboulfidaa N., Bouslikhane M., Fassi-Fihri O. (2017). A Century Spent Combating Rabies in Morocco (1911-2015): How Much Longer?. Front. Vet. Sci..

[15] Nguyen H.T.T., Afriyie D.O., Tran C.H., Dang A.D., Tran D.N., Dang T.Q., Otsu S., Urabe M.I., Pham T.N., Nguyen H.T. (2019). Progress towards rabies control and elimination in Vietnam. Rev. Sci. Tech..

[16] Ghosh S., Rana M.S., Islam M.K., Chowdhury S., Haider N., Kafi M.A.H., Ullah S.M., Shah M.R.A., Jahan A.A., Mursalin H.S. (2020). Trends and clinico-epidemiological features of human rabies cases in Bangladesh 2006–2018. Sci. Rep..

[17] Nihal P.D.B., Dangolla A., Hettiarachchi R., Abeynayake P., Stephen C. (2019). Surveillance Opportunities and the Need for Intersectoral Collaboration on Rabies in Sri Lanka. J. Vet. Med..

[18] Taylor L.H., Knopf L. (2015). Surveillance of Human Rabies by National Authorities—A Global Survey. Zoonoses Public Health.

[19] Banyard A.C., Horton D.L., Freuling C., Müller T., Fooks A.R. (2013). Control and prevention of canine rabies: The need for building laboratory-based surveillance capacity. Antivir. Res..

[20] Kanda K., Jayasinghe A., Jayasinghe C., Yoshida T. (2021). Public health implication towards rabies elimination in Sri Lanka: A systematic review. Acta Trop..

[21] Thomas L.F., Rushton J., Bukachi S.A., Falzon L.C., Howland O., Fèvre E.M. (2021). Cross-Sectoral Zoonotic Disease Surveillance in Western Kenya: Identifying Drivers and Barriers Within a Resource Constrained Setting. Front. Vet. Sci..

[22] Fooks A.R., Banyard A.C., Horton D.L., Johnson N., McElhinney L.M., Jackson A.C. (2014). Current status of rabies and prospects for elimination. Lancet.

[23] Scott T.P., Coetzer A., de Balogh K., Wright N., Nel L.H. (2015). The Pan-African Rabies Control Network (PARACON): A unified approach to eliminating canine rabies in Africa. Antivir. Res..

[24] Coetzer A., Scott T.P., Amparo A.C., Jayme S., Nel L.H. (2018). Formation of the Asian Rabies Control Network (ARACON): A common approach towards a global good. Antivir. Res..

[25] World Health Organization (2013). WHO Expert Consultation on Rabies: Second Report.

[26] Knobel D.L., Cleaveland S., Coleman P.G., Fèvre E.M., Meltzer M.I., Miranda M.E., Shaw A., Zinsstag J., Meslin F.X. (2005). Re-evaluating the burden of rabies in Africa and Asia. Bull. World Health Organ..

[27] US Centers for Disease Control and Prevention Is Rabies in Your State?. https://www.cdc.gov/rabies/location/usa/surveillance/.

[28] Vigilato M.A., Clavijo A., Knobl T., Silva H.M., Cosivi O., Schneider M.C., Leanes L.F., Belotto A.J., Espinal M.A. (2013). Progress towards eliminating canine rabies: Policies and perspectives from Latin America and the Caribbean. Philos. Trans. R. Soc. Lond. B Biol. Sci..

[29] Ueki H. (2007). Tokyo Rabies Epidemic Journal (Reprint Edition).

[30] Ministry of Health, Labour and Welfare of Japan [MHLW] Rabies. https://www.mhlw.go.jp/bunya/kenkou/kekkaku-kansenshou10/.

[31] MHLW Annual Trend of the Number of Registered and Vaccinated Dogs in 1955–2019. https://www.mhlw.go.jp/bunya/kenkou/kekkaku-kansenshou10/02.html.

[32] Velasco-Villa A., Escobar L.E., Sanchez A., Shi M., Streicker D.G., Gallardo-Romero N.F., Vargas-Pino F., Gutierrez-Cedillo V., Damon I., Emerson G. (2017). Successful strategies implemented towards the elimination of canine rabies in the Western Hemisphere. Antivir. Res..

[33] Global Alliance for Rabies Control Stepwise Approach towards Rabies Elimination (SARE). https://rabiesalliance.org/tools/planning-tools/sare.

